# Renal angiotensin II type 1 receptor expression and associated hypertension in rats with minimal SHR nuclear genome

**DOI:** 10.1002/phy2.104

**Published:** 2013-10-20

**Authors:** Jason A Collett, Anne K Hart, Elaine Patterson, Julie Kretzer, Jeffrey L Osborn

**Affiliations:** 1 Department of Biology, University of KentuckyLexington, Kentucky; 2 Department of Anatomy and Neurobiology, University of Texas Health Science Center at HoustonHouston, Texas; 3 College of Medicine, University of KentuckyLexington, Kentucky

**Keywords:** Angiotensin receptors, genetic hypertension, renin–angiotensin system

## Abstract

Angiotensin II (AII) has been linked as a causal factor in several experimental models of hypertension (HT) including Okamoto spontaneously hypertensive rats (SHR). The transmission and expression of AII type 1 receptors (AT_1_r) in SHR and the development of genetic HT remain unknown. It is hypothesized that tissue-specific expression of renin–angiotensin system (RAS) genes derived from SHR are linked to HT in offspring of SHR crossed with Brown Norway (BN) rats. Hypertensive female progeny of BN/SHR matings was backcrossed with founder BN males to generate the F1 and five backcross generations (BN/SHR-mt^SHR^). Progeny were phenotyped according to normotension (NT: systolic arterial pressure [SAP] ≤ 124 mmHg), borderline hypertension (BHT: 124 ≤ SAP < 145 mmHg), and HT (SAP ≥ 145 mmHg). Six generations produced more HT (*n* = 88; 46%) than NT (*n* = 21; 11%) offspring. The mRNA expression of the RAS was evaluated in NT (*n* = 20) and HT (*n* = 20) BN/SHR-mt^SHR^ across several generations. Quantitative real-time polymerase chain reaction analysis of kidney tissue showed increased expression of AII, type 1 receptors (*Agtr1a*) (∼2.5-fold) in HT versus NT rats, while other members of both the renal and systemic RAS pathway were not different. Western blot analysis from kidney homogenates showed that AT_1_r protein levels were higher (*P* < 0.05) in backcross generation 3 (BC3) HT versus NT rats. Evaluation of SAP as a function of AT_1_r expression by linear regression indicated positive correlation (*P* < 0.05) in kidney of BC3 BN/SHR-mt^SHR^ rats. Thus, elevated kidney AT_1_r expression may be involved in the development of HT in BN/SHR-mt^SHR^ rats.

## Introduction

Hypertension (HT) and the chronic elevation of blood pressure (BP) constitute a primary and significant factor in the development of cardiovascular disease. Essential HT is regarded as a multifactorial disease, influenced by both genetic makeup and environmental conditions that can alter genomic expression (National Heart, Lung and Blood Institute, Nihon Gaujutsu Shinkōkai, National Science Foundation 1977; Raizada et al. 1993; Schork et al. 1995). The polygenic nature of HT and its dependence on environmental factors complicate the clear identification of genetic factors that directly increase BP (Joe and Garrett 2005). The rat is a well-established animal model for investigating human HT, with over 25,000 studies reported on HT in rats alone (Kwitek-Black and Jacob 2001). Rat models of spontaneous HT, in particular, the spontaneously hypertensive rat (SHR), have been critical to our understanding of BP control and the pathophysiology of HT. Significant progress has been made to map the genes involved in BP regulation in these animal models. Nuclear and mitochondrial genome scans have been carried out in rats, mice, and humans and have revealed several potential genomic regions that may contain genes involved in the pathogenesis of spontaneous HT (Rapp 2000). Molecular genetic analysis proves that BP regulation is polygenic, and there is good evidence for several BP-related quantitative trait loci (QTLs) on nearly every rat chromosome (Hilbert et al. 1991; Deng et al. 1994; Rapp et al. 1994; Schork et al. 1995; Doris 2002; Hopkins and Hunt 2003). Linkage analyses of populations derived from the SHR show potential BP QTLs at numerous loci; however, the precise nature of the genetic mechanisms underlying essential HT remains unanswered.

Significant data from our laboratory and others have documented that angiotensin II (AII) and the renin–angiotensin system (RAS) play a critical role in maintenance of arterial BP and that this hormonal system is elevated in several experimental models of HT, as well as human essential HT (Cowley 1992; Reinhart et al. 1995; Lenkei et al. 1997; Weir and Dzau 1999; de Gasparo et al. 2000; Lifton et al. 2001; Doris 2002). It has been suggested that the RAS plays a pathogenic role in the development of HT in Aoki–Okamoto SHR. An elevated RAS impacts BP directly via vasoconstriction and sodium retention, as well as indirectly through increased reactive oxygen species (ROS), altering redox signaling and increased sympathetic outflow.

Here, we demonstrate a unique rat colony developed by breeding a hypertensive female Okamoto–Aoki SHR with male, normotensive Brown Norway (BN) rats. Hypertensive female offspring were backcrossed with the original males for five subsequent generations. Despite the dilution of the “hypertensive” nuclear genome, hypertensive phenotype expressed by the founder female was dominantly expressed and maintained across six generations of BN/SHR-mt^SHR^ rats. All progeny, however, have identical mitochondrial genomes. We investigated the tissue-specific mRNA expression of the RAS pathway, including angiotensinogen (*AGT*), renin (*REN*), angiotensin-converting enzyme 1 (*ACE1*), angiotensin-converting enzyme 2 (*ACE2*), and AII, type 1 receptors (*Agtr1a*) in normotensive and hypertensive BN/SHR-mt^SHR^ backcross rats. It is hypothesized that tissue-specific increased RAS expression contributes to heritable HT in BN/SHR-mt^SHR^ rats.

## Material and Methods

### Experimental animals

All experiments were carried out in accordance with the AAALAC Guide to the Care and Use of Laboratory Animals and all protocols were previously approved by the University of Kentucky Institutional Animal Care and Use Committee (UK IACUC). A congenic colony using phenotypic selection was employed. The Aoki–Okamoto SHR/Brown Norway (BN/SHR-mt^SHR^) rat colony was developed by breeding a female SHR (Charles River Labs, Wilmington, MA) with two different normotensive Brown Norway males (BN* and BN∧, respectively; Charles River Labs). Rats were raised in a 12 h light:12 h dark cycle in a climate of 20–22°C from birth. At 3 weeks of age, rats were weaned and transferred to either individual (males) or group (≤3 females of the same litter) solid-wall cages with bedding and were provided a commercial standard rodent chow and tap water ad libitum. Beginning at 10 weeks of age, rats were phenotyped as normotensive, borderline hypertensive (BHT), or hypertensive using tail cuff plethysmography (Kent Scientific, Torrington, CT). Hypertensive female offspring were then backcrossed to the original progenitor BN males for five subsequent generations. All rats in the colony possessed identical mitochondrial genomes, with increasing BN nuclear genome with each subsequent backcross generation. After repeated BP recordings that assured consistent determination of adult arterial pressure, rats were euthanized with an overdose of sodium pentobarbital (60 mg/kg i.p.), immediately decapitated, and organ tissues were rapidly frozen in a solution of dry ice and acetone.

Age- and sex-matched HT (*N* = 20) and normotension (NT) (*N* = 20) animals across six generations of BN/SHR-mt^SHR^ were chosen for RAS mRNA evaluation. Animals in the backcross generation 3 (BC3) were chosen for protein analysis as there were an appropriate number of age- and sex-matched NT and HT rats within a single generation.

### Measurement of arterial pressure

Systolic arterial pressure (SAP) was evaluated in parents and offspring beginning at 10–12 weeks of age. Phenotypes were assigned as normotensive (NT: SAP ≤ 124 mmHg), BHT (125 ≤ SAP < 145 mmHg), or hypertensive (HT: SAP ≥ 145 mmHg). As animals were to be back bred to the founder males in the establishment of the conplastic genome, tail cuff plethysmography was used as a phenotyping methodology only to establish basic individual BP. To minimize stress and improve reliability of BP measurements, several steps were used in the BP recording method that has been previously characterized and published (Kurtz et al. 2005). Rats were exposed and acclimated to the measurement procedures and restraint equipment prior to BP recordings. A dark cover was placed over the restrained animal for the duration of the BP measurement, and BP recordings were performed at the same time each day. All equipments were thoroughly cleaned and disinfected before and after each individual rat to eliminate foreign scent. Animals were moderately warmed to dilate the ventral artery. Arterial pressures were derived from the average results of ≥5 measurements in each recording session. The average BP of ≥5 mmHg separate recording sessions with <5% variability was used to establish the phenotype of each animal. Both systolic and diastolic pressures were obtained and recorded. For purposes of reporting, the systolic pressures were used for the determination of the specific individual phenotype.

### RNA extraction and real-time polymerase chain reaction

Kidney, liver, and lung tissue were harvested from HT and NT rats (*n* = 20 NT; *n* = 20 HT) as described above. Total RNA was extracted by Trizol reagent (Invitrogen, Carlsbad, CA) and purified using RNeasy minicolumns (Qiagen Inc., Valencia, CA) according to the manufacturer's protocol. Possible genomic DNA in total RNAs was digested with RNA-free DNase I (Qiagen Inc.). Concentration and purity of all RNA samples were determined by the Nanodrop ND-1000 spectrophotometer (Nanodrop Technologies, Wilmington, DE). Extracted RNA was reverse transcribed into complementary DNA (cDNA) using qScript cDNA supermix (Quanta Biosciences, Gaithersburg, MD) in a total volume of 20 μLS using a MyCyler Thermal Cycler (Bio-Rad Laboratories, Hercules, CA).

### Quantitative real-time polymerase chain reaction

Quantitative real-time polymerase chain reaction (RT-PCR) was performed on a StepOnePlus real-time PCR system (Applied Biosystems Inc., Foster City, CA). Real-time quantitative PCR amplifications were performed in triplicate in a 96-well plate. For normalization, *GAPDH* was used as the reference gene. Predesigned primers and hydrolysis probes were purchased from Integrated DNA Technologies, Inc. (Coralville, IA). (*Agtr1a*; Primer 1: 5′-CCAGCCATTTTATACCAATCTC-3′, Primer 2: 5′-TCCTGTTCCACCCGATCA-3′, Probe: 5′-/HEX/CAGCTCTGC/ZEN/CACATTCCCTGAGT/3IABkFQ/-3′.) (GAPDH; Primer 1: 5′GTAACCAGGCGTCCGATAC-3′, Primer 2: 5′-GTTCTAGAGACAGCCGCATC-3′, Probe: 5′-/56-FAM/ATCCGTTCA/ZEN/CACCGACCTTCACC/3IABkFQ/-3′). Predesigned TaqMan primers and hydrolysis probes for AGT (Rn00593114_m1), REN (Rn00561847_m1), ACE1 (Rn00561094_m1), and ACE2 (Rn01416293_m1) were purchased from Life Technologies (Foster City, CA). Relative gene expression was calculated using the Comparative C_T_ method (2^−^▵▵CT). Primers and probes were verified and operated at similar efficiencies.

### Membrane protein extraction

Whole kidney tissues were placed in an ice-cold buffer solution containing 1 mol/L Tris, 5 mol/L NaCl, 0.5 mol/L ethylenediaminetetraacetic acid, 100% Brij 96/97, and 10% NP40 with 0.3% protease inhibitor leupeptin (50 μmol/L), aprotinin (50 nmol/L), and pepstatin (1 μmol/L). Tissue samples were immediately homogenized (PowerGen® 125 Homogenizer; Fisher Scientific, Pittsburgh, PA) at 4°C for ∼15 sec. After complete homogenation, samples were loaded into a centrifuge (Heraeus Biofuge 13; Baxter Scientific, Deerfield, IL) and spun at 13,000 rpm for 10 min at 4°C. Protein concentration was determined for each sample using colorimetric assay according to Lowry et al. (1951).

### Western blot analysis of AT1 receptor

Kidney protein samples were electrophoretically separated on 4–10% sodiumdodecyl sulfate polyacrylamide gel electrophoresis (SDS-PAGE) gels at 150 V/50 mA for 1 h. Separated proteins were transferred by electroelution (200 V, 1–2 h) to polyvinylidene difluoride (PVDF) membranes (0.45 μm; Millipore, Bedford, MA). Molecular weight markers (∼10–190 kDa; Benchmark Prestained Protein Ladder, Invitrogen) were used to estimate molecular mass. Blots were blocked with 5% bovine serum albumin in Tris-buffered saline. Blots were incubated with primary antibodies AII type 1 receptors (AT_1_r) (1:200, sc-81671) and β-tubulin (loading control; 1:200, sc-23949; Santa Cruz Biotechnology Inc., Santa Cruz, CA) at 25°C for 2 h. Blots were washed with Tris-buffered saline/0.1% Tween-20 (TBST) and then exposed to secondary antibody conjugated to horseradish peroxidase (1:2000, sc-2005; Santa Cruz Biotechnology Inc.) at 25°C for 2 h. Detection of specific proteins was accomplished using enhanced chemiluminescence (SuperSignal West Pico; Thermo Scientific, Rockford, IL) according to the manufacturer's instructions, and blots were exposed to BioMax Light Autoradiography film (Kodak #1788207, Rochester, NY). Densitometric results were reported as integrated values (area density of band) and expressed as a ratio of AT_1_r to loading control (β-tubulin). Results were then compared between phenotypic groups. Lanes lacking protein were subject to AT_1_r and β-tubulin primary antibody to verify antibody selectivity. Densitometry reflects mean ± SEM of all samples.

### Data analysis

Blood pressures as well as tissue AT_1_r protein expression between NT and HT BN/SHR-mt^SHR^ rats were analyzed using a Student's *t*-test. Renal *AGT*, *REN*, *ACE1*, *ACE2*, and *Agtr1a* mRNA expression levels between HT and NT BN/SHR-mt^SHR^ rats were analyzed using Mann–Whitney *U*-test comparisons. The 0.05 level of probability was utilized as the criterion of significance. All statistical analyses were performed using GraphPad Prism 4 (GraphPad Software, Inc., La Jolla, CA).

## Results

### Arterial pressure phenotyping of BN/SHR-mt^SHR^ colony

**Figure 1:**
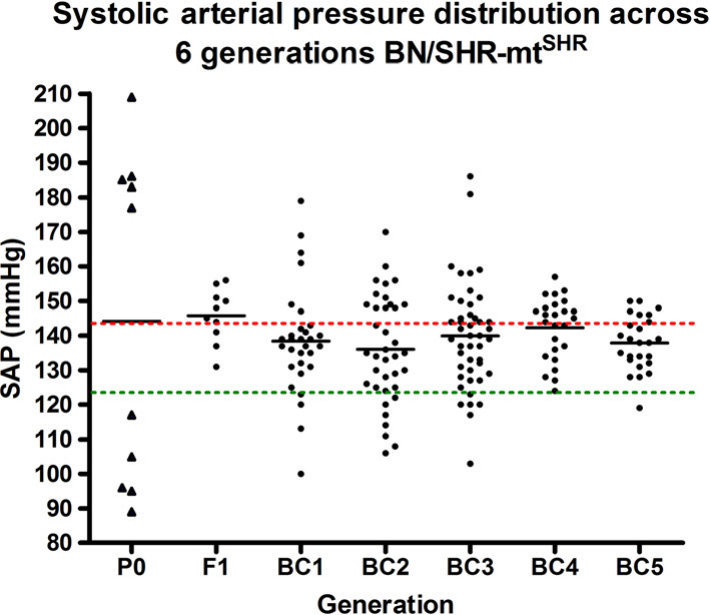
Six generations of BN/SHR-mt^SHR^ rats with corresponding average systolic arterial pressure (SAP) values. Three distinct populations persisted throughout all six generations, with hypertension being dominantly expressed and maintained.

Systolic arterial pressure phenotypes were assessed weekly beginning at 10–12 weeks of age according to the following arterial BP parameters: NT: SAP ≤ 124 mmHg; BHT: 125 mmHg < SAP < 145 mmHg; or HT: SAP > 145 mmHg. Progenitor female SHR had a SAP of 188 mmHg and progenitor BN males had SAPs of 105 (BN*) and 103 (BN∧) mmHg. The BN*/SHR-mt^SHR^ cross/backcross produced six generations, yielding 94 total offspring, with 42.6% (*n* = 40) expressing the hypertensive phenotype, 42.6% (*n* = 40) expressing the BHT phenotype, and only 14.9% (*n* = 14) expressing the normotensive phenotype. The BN^∧^/SHR-mt^SHR^ cross/backcross also produced six generations, yielding 71 total offspring, with 52.1% (*n* = 37) expressing the hypertensive phenotype, 39.4% (*n* = 28) expressing the BHT phenotype, and only 8.5% (*n* = 6) expressing the normotensive phenotype. Together, the six total generations produced 190 offspring, with 110 (58%) female and 80 (42%) male offspring. There were significantly more hypertensive (*n* = 88; 46%) than normotensive offspring (*n* = 21; 11%), while a large number of individuals expressed the intermediate phenotype (*n* = 81; 43%). There were no differences in SAP between male and female offspring at any generation. Furthermore, comparison of systolic, diastolic, and mean arterial pressures of male and female offspring across all backcross generations did not demonstrate any gender differences in arterial pressures. HT was dominantly expressed and maintained across all six offspring generations of BN/SHR-mt^SHR^ rats (Fig. 1).

### Renal *Agtr1a* mRNA expression is higher in hypertensive BN/SHR than in normotensive BN/SHR-mt^SHR^ rats

**Figure 2:**
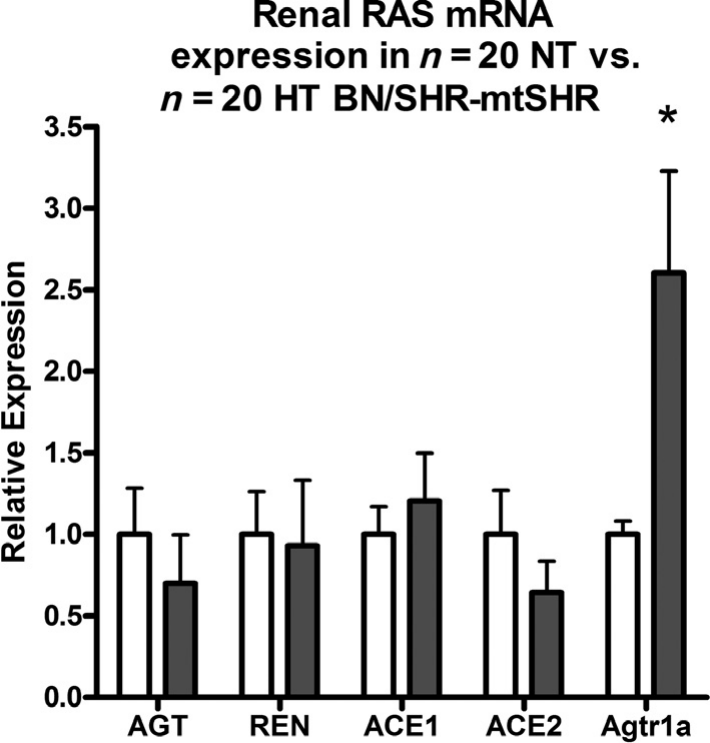
Quantitative real-time PCR of renal *AGT*, *REN*, *ACE1*, *ACE2,* and *Agtr1a* in age- and sex-matched hypertension (HT) and normotension (NT) rats. Data were normalized to *GAPDH* RNA from the same samples (NT *n* = 20; HT *n* = 20). There was no difference in *AGT*, *REN*, *ACE1,* and *ACE2* expression of HT rats compared to NT rats across 6 generations. *Agtr1a* mRNA levels were ∼2.5× higher in HT rats compared to NT rats.

The mRNA levels of *AGT*, *REN, ACE1*, and *ACE2* and *Agtr1a* in renal tissue were evaluated in HT and NT BN/SHR-mt^SHR^ rats (*n* = 20 NT; *n* = 20 HT). Animals exhibiting the most extreme phenotypes were chosen for mRNA expression analysis. *AGT*, *REN*, *ACE1*, and *ACE2* mRNA levels were not different between NT and HT BN/SHR-mt^SHR^ rats. Renal *Agtr1a* was increased by ∼2.5-fold (*P* < 0.05) in HT compared to NT BN/SHR-mt^SHR^ rats (Fig. 2). Expression is reported as relative expression using the 2^−ΔΔCT^ method. NT values were normalized to 1. *GAPDH* was used as the reference gene and data are expressed as a ratio of specific mRNA expression and *GAPDH*.

### Systemic RAS mRNA expression is not different in hypertensive compared to normotensive BN/SHR-mt^SHR^ rats

**Figure 3:**
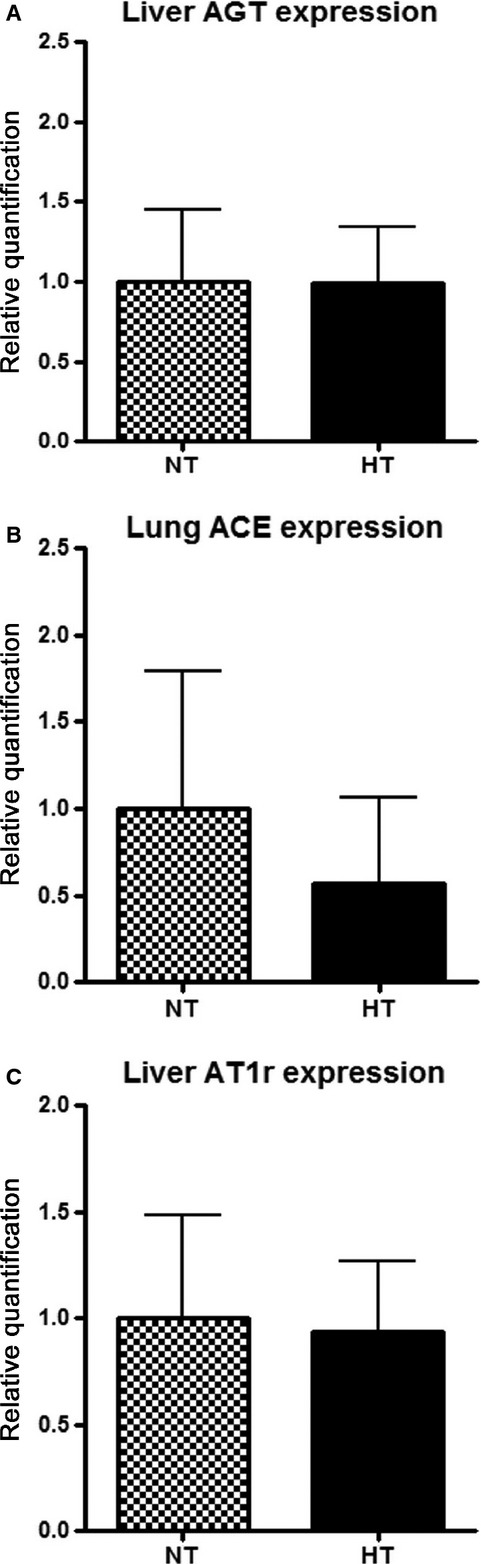
Quantitative real-time PCR of liver *AGT* (A), lung *ACE1* (B), and liver *Agtr1a* (C) in BC3 HT and NT rats. Data were normalized to *GAPDH* RNA from the same samples (NT *n* = 6; HT *n* = 5). There was no difference in liver *AGT,* lung *ACE1*, *or* liver *Agtr1a* mRNA expression in HT BC3 rats compared to NT BC3 rats.

The mRNA levels of liver *AGT*, *Agtr1a*, and lung *ACE1* were evaluated in HT and NT BN/SHR-mt^SHR^ rats (*n* = 20 NT; *n* = 20 HT). Animals exhibiting the most extreme phenotypes were chosen for mRNA expression analysis. Liver *AGT*, *Atgr1a*, and lung *ACE1* mRNA levels were not different between NT and HT BN/SHR-mt^SHR^ rats (Fig. 3A–C). Expression is reported as relative expression using the 2^−ΔΔCT^ method. NT values were normalized to 1. *GAPDH* was used as the reference gene and data are expressed as a ratio of specific mRNA expression and *GAPDH*.

### Renal AT1 receptor protein expression is higher in hypertensive BN/SHR-mt^SHR^ than in normotensive BN/SHR-mt^SHR^ rats

**Figure 4:**
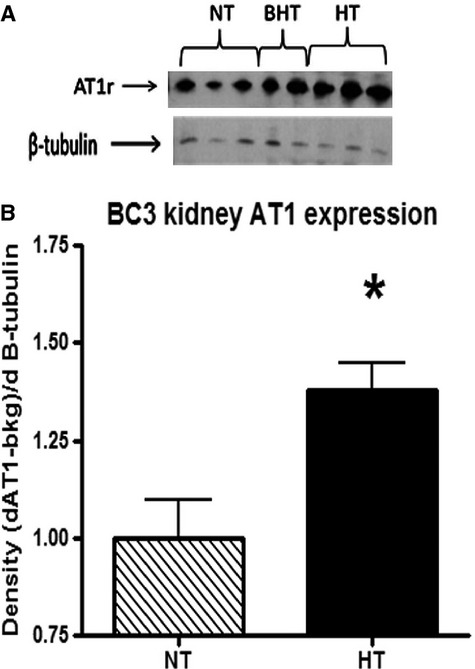
Semiquantitative immunoblotting of whole kidney tissue homogenates of BC3 HT and NT rats. Representative Western blots of AT1r and β-tubulin are shown in A. The results of Western blot analysis indicate that AT1r protein expression was higher in both BHT and HT rat kidneys, than in NT rat kidneys (*P < 0.05). A summary figure with the ratio of AT1r and β-tubulin with each corresponding phenotype is shown in B.

Western blot analysis of AT_1_ receptor protein from whole kidney homogenates using the monoclonal antibody showed that AT_1_ receptor protein levels were not different (*P* > 0.05) between BHT and HT F1 BN/SHR-mt^SHR^ rats. Whole kidney homogenates (Fig. 4) using the same monoclonal antibody showed that receptor protein levels were significantly higher (*P* < 0.05) in BC3 HT rats compared to NT rats. The ratio of renal AT_1_ to β-tubulin densitometric signals was 1.000 ± 0.097 versus 1.379 ± 0.06975.

**Figure 5:**
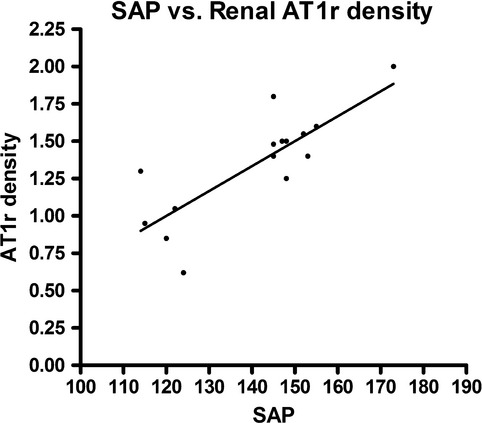
Regression analysis between SAP and AT1r protein expression in whole kidney homogenates of BC3 rats. Average systolic arterial pressure SAP and AT_1_r protein expression were positively correlated (*r*^2^ = 0.6502, *P < 0.05).

Regression analysis was also performed, indicating a positive correlation (*r*^2^ = 0.6502, *P* < 0.05) between SAP and AT_1_ receptor protein expression in kidney (Fig. 5) BC3 BN/SHR-mt^SHR^ rats.

## Discussion

The main findings of this study are as follows: (1) elevated arterial pressure is dominant and maintained across six generations of BN/SHR-mt^SHR^ rats, despite the increasing nuclear genomic contribution of the NT male donor rats; (2) hypertensive BN/SHR-mt^SHR^ rats across six generations exhibit elevated renal *Agtr1a* mRNA expression compared to NT BN/SHR-mt^SHR^ rats, while expression of renal *REN*, liver *AGT* and *Agtr1a*, and lung *ACE1* was not different; (3) hypertensive BN/SHR-mt^SHR^ in the BC3 generation exhibit an elevated AT_1_r protein expression in kidney compared to NT BN/SHR-mt^SHR^ rats; and (4) increased AT_1_r expression is positively correlated with elevated SAP in BC3 BN/SHR-mt^SHR^ rats. Taken together, these results suggest that HT is dominant in the presence of increasing normotensive “loci” and that altered expression of AT_1_r, but not other aspects of renal or systemic RAS, may underlie heritable HT.

The SHR has been the most widely studied genetic model of essential HT in the past four decades (Pinto et al. 1998). Neurogenic in nature, the underlying mechanisms of the onset and manifestation of HT in the SHR remain to be fully elucidated. In this experimental paradigm, an attempt to isolate genomic regions from which HT derives in a “conplastic” colony using phenotypic selection was employed. In these rats, the nuclear genome of the sixth generation (BC5) offspring is comprised of ∼96.9% original BN males, and only 1.6% of original donor SHR. The dilution of the hypertensive genome had seemingly little effect on the manifestation of the hypertensive phenotype from generation F1 through backcross five (Fig. 2). It is clear, however, that the magnitude of the HT was never as high in the offspring, as in the progenitor SHR even from the initial F1 generation. In fact, there is approximately a 30 mmHg difference between the average SAP of the SHR and F1 generation offspring. However, from the F1 generation forward, the magnitude of elevated BP did not decline toward the BN BP in any of the subsequent offspring generations. Thus, although a significantly increasing influence of the BN genome is transmitted following genetic mixing with SHR of subsequent backcross generations, the impact of the SHR genome was fully maintained for six consecutive generations (F1-BC5) following the initial mating.

Since the discovery of “renin” in 1898 and subsequent recognition that AII is pressor, the RAS has been extensively studied and remains a major candidate as a causative factor in elevated arterial pressure and the pathogenesis of HT (Tigerstedt and Bergman 1898; de Gasparo et al. 2000; Carey and Siragy 2003). The AII, type 1 receptor has been extensively evaluated in rodents and has subsequently become a target as a causal factor in the development of essential HT. Reja et al. (2006) showed that gene expression levels of AT_1_r, extracellular signal-regulated kinase 2, and phosphatidylinositol 3-kinase were significantly higher in the paraventricular nucleus of the hypothalamus, rostral ventrolateral medulla, and adrenal medulla in SHR compared to normotensive Wistar Kyoto (WKY) rats. Raizada et al. (1993) showed that AT_1_r mRNA was higher in the brains of the SHR compared to normotensive WKY rats. Furthermore, Gyurko et al. (1993) showed that antisense inhibition of AT_1_ receptor mRNA in the brain reduces the magnitude of HT in adult SHR. Data from our study support the notion that elevated AT_1_ receptors may play a role in SHR-derived elevated arterial pressure. Hypertensive BN/SHR-mt^SHR^ rats across several generations exhibit elevated renal-specific *Agtr1a* mRNA expression, while expression of other renal RAS components, as well as liver *AGT* and lung *ACE1* was not different (Figs. 2, 3). In the BC3 generation, where the nuclear genome of the original SHR accounts for only ∼6.2%, HT rats had significantly higher AT_1_r and protein expression than normotensive rats in kidney tissue (Fig. 4). Furthermore, average SAP and tissue expression of AT_1_r were positively correlated (Fig. 5), indicating that tissue-specific expression of AT_1_r expression may critically impact the development and maintenance of SHR HT. Increased AT_1_r protein could potentially have drastic effects on the cardiovascular system, including the pathogenesis of HT, and seems to play a role in the development of HT in BN/SHR-mt^SHR^ rats. AII's effect in the kidney would be exacerbated, increasing proximal tubular sodium reabsorption and decreasing renal blood flow. Recently, commercially available AT_1_r antibodies have come under scrutiny for lack of specificity, and therefore may lead to erroneous results (Herrera et al. 2013). It is critical to note here, however, that the antibody used for this study has not previously been identified as nonspecific, and that our mRNA expression data fully corroborates the protein data that AT1r expression is elevated in kidney of BN/SHR-mt^SHR^ backcross rats.

With the abundance of knowledge on the role of both circulating and tissue RAS in BP control, it is hypothesized that genetic variability in one or more of the RAS components could account for the pathogenesis of HT. Common variants of the RAS genes, including those coding for angiotensinogen and angiotensin-converting enzyme were some of the first to be associated with altered BP control (Norton et al. 2010). Several linkage analysis and genome-wide association studies have been performed in both rodents and humans with regard to RAS genes, producing variable results (Rigat et al. 1990; Jeunemaitre et al. 1992; Bonnardeaux et al. 1994; Schmidt et al. 1997; Rothermund and Paul 1998; Kainulainen et al. 1999; Tomino et al. 1999; Zhu et al. 2000; Baudin 2002). Results from this study highlight a renal-specific elevation in a single gene of the RAS, indicating that genomic polymorphisms at this allele are not causal to the manifestation of the disease. Gene expression and subsequent protein synthesis may be equally, if not more important than heritable nucleotide differences in elucidating the causes of complex, multigenic diseases. Data from this study indicate that AT_1_r mRNA and receptor protein expression may have a significantly more important role than other aspects of RAS in heritable HT.

Due to the breeding paradigm (i.e., backcrossing hypertensive females with founder males), the influence of the mitochondrial genome (female transmission) on the pathophysiology of HT should be taken into significant consideration. These results provide strong evidence for the dominance of loci within the SHR genome that are highly resistant to increasing normotensive influence of the BN genome. At present, there are few data that identify the specific genes located in these “SHR dominant” regions. However, it is likely that these genomic regions contain numerous BP-controlling gene loci (Lowry et al. 1951; Rapp 2000; Kaschina and Unger 2003). As hypertensive females were phenotypically selected for backcross, all offspring (F1, BC2-BC5) should have identical mitochondrial genomes, barring any mutation(s). Mitochondrial dysfunction has recently been implicated in a wide variety of genetic disorders (Wallace 1999; Taylor and Turnbull 2005). Alterations in mitochondrial function are observed in conjunction with the development of HT in rodents and humans (Pravenec et al. 2007; Chan et al. 2009; Kumarasamy et al. 2010). The genetic association of mtDNA variants (Wilson et al. 2004; Kumarasamy et al. 2010) and tRNA mutations (Pravenec et al. 2007; Li et al. 2009; Liu et al. 2009; Kumarasamy et al. 2010) with type 2 diabetes and HT directly implicated mitochondrial defects to the etiology of cardiovascular disease and metabolic syndrome. Mitochondrial integrity and potential dysfunction is currently being evaluated in the BN/SHR-mt^SHR^ rats.

A potentially critical avenue in investigating the underlying mechanisms of heritable HT in BN/SHR-mt^SHR^ is the relationship between the RAS and mitochondrial dysfunction. The role played by AII in developing mitochondriopathy has been advanced recently by Benigni et al. (2009). Deletion of the *Agtr1a* gene resulted in the reduced age-related cardiorenal complications, improved mitochondrial biogenesis, and increased longevity in mice. Additionally, treatment with antioxidants, mitochondrial superoxide dismutase mimetics, and AT_1_r blockers decreased vascular O_2_ production and attenuated development of HT in SHR (Park et al. 2002; Rodriguez-Iturbe et al. 2003; Shokoji et al. 2003). de Cavanagh et al. (2006) have demonstrated that oxidative stress is associated with mitochondrial dysfunction in SHR, and that this dysfunction is attenuated with AT1r blockade with losartan. Taken together, there is significant evidence of mtDNA mutations and/or altered mitochondrial genetic expression is influenced, at least in part, by the RAS, and that this relationship may play a significant role in the development of HT.

In summary, the present results indicate that HT is dominantly expressed in BN/SHR-mt^SHR^ rats, and that elevated arterial pressure is positively correlated with the upregulation of kidney AT_1_r mRNA and protein expression, whereas other aspects of the local and systemic RAS pathways are not different. It has been reported that AT_1_r mRNA is regulated in a tissue-specific manner that is distinct among the other components of the RAS, and potentially independent of changes in circulating AII (Sechi et al. 1996). Our current study supports this finding, as well as adds insight into the heritability and expression of different aspects of RAS. The maintenance of HT under conditions where the proportion of the female progenitor SHR mitochondrial genome remains intact, nuclear genome is continually reduced by increasing amounts of normotensive progenitor BN genome strongly suggests a major linkage of the SHR genes to the development of HT. While the origins of this SHR-derived HT remain unknown, strong physiological evidence for a major neurogenic and RAS components have been reported. Given that the maintenance of HT in the F1 and subsequent backcross generations, the encoding and linkage of hypertensive loci may be directly related to genomic components of the female SHR mitochondrial genome.

The presence and abundance of AT_1_r in the kidney appear to be related to the propagation of the HT phenotype in BN/SHR-mt^SHR^. This work adds to the body of evidence that quantitative variations in gene expression at loci encoding components of the renin–angiotensin–aldosterone system may be genetically linked to or associated with physiological alterations in BP. Although the RAS pathway has been a major therapeutic target for decades, understanding the heritability of expression of these targets can help switch the focus from treatment to prevention. The role of AT1 receptors in individual tissues and their differential expression provide valuable information on how personalized therapy can be used to better treat or prevent cardiovascular disease in the future.
